# The COVID-19 nephrology compendium: AKI, CKD, ESKD and transplantation

**DOI:** 10.1186/s12882-020-02112-0

**Published:** 2020-10-27

**Authors:** Sam Kant, Steven P. Menez, Mohamed Hanouneh, Derek M. Fine, Deidra C. Crews, Daniel C. Brennan, C. John Sperati, Bernard G. Jaar

**Affiliations:** 1 Department of Medicine, Johns Hopkins School of Medicine, 5601 Loch Raven Boulevard, Suite 3 North, Baltimore, MD 21205 USA; 2 Nephrology Center of Maryland, Baltimore, MD USA; 3 Welch Center for Prevention, Epidemiology and Clinical Research, Johns Hopkins University, Baltimore, MD USA; 4 Department of Epidemiology, Johns Hopkins Bloomberg School of Public Health, Baltimore, MD USA

**Keywords:** AKI, CKD, ESKD, Transplantation, Disparity, CoVID-19

## Abstract

The pandemic of coronavirus disease 2019 (CoVID-19) has been an unprecedented period. The disease afflicts multiple organ systems, with acute kidney injury (AKI) a major complication in seriously ill patients. The incidence of AKI in patients with CoVID-19 is variable across numerous international studies, but the high incidence of AKI and its associated worse outcomes in the critical care setting are a consistent finding. A multitude of patterns and mechanisms of AKI have been elucidated, and novel strategies to address shortage of renal replacement therapy equipment have been implemented. The disease also has had consequences on longitudinal management of patients with chronic kidney disease and end stage kidney disease. Kidney transplant recipients may be especially susceptible to CoVID-19 as a result of immunosuppression, with preliminary studies demonstrating high mortality rates. Increased surveillance of disease with low threshold for testing and adjustment of immunosuppression regimen during acute periods of illness have been recommended.

## Background

The coronavirus disease 2019 (CoVID-19) pandemic has strained medical systems globally. The disease results from infection with severe acute respiratory syndrome coronavirus-2 (SARS-CoV-2), and it results in multi-organ injury including the kidneys. The disease has unique implications for patients developing acute kidney injury (AKI), as well as patients with chronic kidney disease (CKD) or end stage kidney disease (ESKD) and kidney transplant recipients (KTR). In this review, we provide a comprehensive overview of the effect of CoVID-19 on these aspects of kidney disease.

We elucidate the epidemiology and associated clinical characteristics of AKI by analyzing various studies that have reported outcomes related to AKI and renal replacement therapy (RRT). We also discuss the mechanisms and patterns of AKI. In the CKD and ESKD sections we discuss the controversy of renin angiotensin system blockade, along with recommendations for managing patients on chronic dialysis in the era of CoVID-19. Kidney transplantation requires particular emphasis during the pandemic due to concern for increased susceptibility to infection. The segment on kidney transplantation incorporates current outcomes of kidney transplant patients with CoVID-19 and recommendations for management of immunosuppression. Finally, we highlight a need for consideration of how the CoVID-19 pandemic is impacting socially disadvantaged populations known to experience worse outcomes in kidney disease.

## Main text

### Acute Kidney injury

AKI was first recognized as a complication of the severe acute respiratory syndrome novel coronavirus (SARS-CoV) in 2003 [1–3]. Based on studies at the time, AKI developed in 6.7% of patients at a median duration of 20 days (range 5–48 days) after onset of viral infection, with 30% requiring renal replacement therapy [4]. Mortality rates of up to 70% in patients with AKI have been reported. In both SARS-CoV and SARS-CoV-2, age, acute respiratory distress syndrome, and AKI were independent predictors of mortality [3, 5].

With respect to SARS-CoV-2, at the time of writing this review, numerous studies have reported a wide incidence of AKI. Three large retrospective studies, involving more than 1000 patients each from China, United Kingdom, and United States, have reported the development of AKI in 0.5–46% of patients [6–8].

#### Epidemiology & Clinical Characteristics

Currently available studies evaluating patient characteristics and disease course are from China, Europe and United States.

AKI appears to be more common in the elderly, males, and those with higher body mass index (median BMI ~ 30 for patients with AKI) [7, 9–11]. Patients with co-morbidities such as CKD, hypertension, coronary artery disease, heart failure, diabetes mellitus, and peripheral arterial disease were more likely to develop AKI [7, 10]. Non-survivors were more likely to have CKD and elevated creatinine during admission, with CKD being an independent predictor of AKI stage 3 (KDIGO criteria) [7, 12].

#### China

Most studies evaluating rates of AKI from China are from single hospitals, with a range of 52–1392 patients for a period of 2–9 weeks [8, 12–23] (Table 1). Reported rates of AKI vary between 0.5–50% [8, 12, 23, 32]. The heterogeneity in rates may be attributable to different definitions for AKI, variable cohort size, and inclusion of patients from different care settings (e.g., all hospitalized vs intensive care unit). The elderly, patients with comorbidities such as hypertension and cardiovascular disease, and ICU patients were more likely to develop AKI. A minority of patients were reported to have pre-existing CKD.

**Table 1:** Studies with demographics and outcomes in patients with COVID-19

**China**										
Study	N (setting)	Female (%)	Median age, years	History of CKD, n (%)	History of CVD, n (%)	Incidence of AKI, n (%)	RRT, n (%)	Mechanical Ventilation, n (%)	Mortality, n (%)	Salient Findings
Yang [15] ^a^	52 (I)	33	52	NR	5 (10%)	15 (29%)	9 (17%)	37 (71%)	32 (61.5%)	–
Wu [17]	80 (G + I)	51.2	46.1	1 (1.2%)	25 (31.3%)	2 (2.5%)	1 (1.2%)	0	0	–
Xia ^a^	81 (G + I)	33	67	3 (3.7%)	28 (35%)	41 (50%) Stage 1: 27% Stage 2: 31% Stage 3: 42%	8 (10%)	66 (80%)	60 (75%)	The primary pathological findings were those of acute tubular injury. Nucleic acid tests and immunohistochemistry failed to detect the virus in kidney tissues. Older age and serum IL-6 levels were risk factors of AKI. KDIGO stage 3 AKI independently predicted death.
Diao [18]	85 (G + I)	43.5	NR	5 (6%)	19 (22.3%)	23 (27%)	NR	NR	NR	AKI likely in elderly patients with comorbidities (HTN, CVD).
Chen [19]	99 (G + I)	32.3	55.5	NR	40 (40%)	3 (3%)	9 (9%)	13 (13%)	11 (11%)	–
Wang [14] ^a^	138 (G + I)	45.7	56	4 (2.9%)	27 (19.5%)	5 (3.6%)	2 (1.5%)	17 (12.3%)	6 (4.3%)	26% required ICU treatment.
Zhou [12] ^a^	191 (G + I)	38	56	2 (1%)	15 (8%)	28 (15%)	10 (5%)	32 (17%)	54 (28.2%)	Non-survivors were likely to be elderly, have comorbidities (CVD, HTN, CKD), or elevated creatinine.
Cao [20]	198 (G + I)	49	50.1	NR	12 (6%)	10 (5.3%)	NR	NR	NR	ICU admissions were more likely to have elevated BUN/creatinine, hyponatremia, CVD.
Zhang [21] ^a^	221 (G + I)	51	55	6 (2.7%)	37 (17%)	10 (4.5%)	5 (2.3%)	26 (12%)	12 (5.4%)	Older patients had higher risk of AKI, ARDS and acute cardiac dysfunction. Patients with severe CoVID likely to have higher BUN/creatinine.
Xiao [16] ^a^	287 (G + I)	44.3	62	5 (2%)	33 (12%)	55 (19%) AKI stage 1: 14.3% AKI stage 2&3: 4.9%	NR	NR	19 (6.6%)	Patients with AKI likely to be older, with HTN, cerebrovascular disease, and likely to present with hypoxia. Patients with AKI also had higher levels of WBC counts, total bilirubin, CK and AST. AKI associated with lower discharge rates and higher mortality.
Pei [22]^a^	333(G + I)	45.3	56.3	NR	NR	35 (10.5%)	6 (0.1%)	NR	29 (8.3%)	Logistic regression analyses showed that severity of pneumonia was associated with lower odds of proteinuric or hematuric remission and recovery from AKI.
Cheng [13] ^a^	701 (G + I)	47.6	63	14 (2%)	NR	36 (5.1%)	NR	97 (13.4%)	113 (16.4%)	Elevated baseline serum creatinine, elevated baseline blood urea nitrogen, AKI stage 1/2/3, proteinuria 1+/2+/3+, and hematuria 1+ were independent risk factors for death.
Guan [7] ^a^	1099 (G + I)	41.9	47	8 (0.7%)	42 (4%)	6 (0.5%)	9 (0.8%)	25 (2.3%)	15 (1.4%)	–
**United Kingdom and France**										
**Study**	**N (setting)**	**Female (%)**	**Median age, years**	**History of CKD, n (%)**	**History of CVD, n (%)**	**Incidence of AKI, n (%)**	**RRT, n (%)**	**Mechanical Ventilation, n (%)**	**Mortality, n (%)**	**Salient Findings**
ICNARC [6]^a^	6143 (I)	28.7%	60	126 (1.6%)	32 (0.4%)	NR	1442 (23.4%)	4287 (70%)	2872 (46.8%)	71% patients on RRT died in ICU.
Rubin [24]^a^	71 (I)	23%	61.2	4 (6%)	21 (30%)	57 (80%) Stage 1: 28% Stage 2: 28% Stage 3: 24%	10 (14%)	55 (71%)	4 (5.6%)	At day 21, 64% of patients had recovered from AKI, and 11% were RRT dependent.
Portolés [25]	1603 (G + I)	40	64	144 (9%)	561 (35%)	336 (21%)	17 (1%)	NR	197 (12.3%)	A prospective cohort study showing in-hospital AKI associated with high mortality
ISARIC [26]	20,133 (I)	40.1%	72.9	2830 (16.2%)	5469 (31%)	NR	NR	618 (37%)	NR	Higher proportion of patients had CKD, with a multivariate HR of 1.28 for death.
**United States**										
**Study**	**N (setting)**	**Female (%)**	**Median age, years**	**History of CKD, n (%)**	**History of CVD, n (%)**	**AKI Incidence, n (%)**	**RRT, n (%)**	**Mechanical Ventilation, n (%)**	**Mortality, n (%)**	**Salient Findings**
Arentz [27] ^a^	21 (I)	48	70	10 (47.6%)	9 (42.9%)	4 (19%)	NR	15 (71%)	11 (52.4%)	2 patients with ESKD.
Mohamed [9] ^a^	575 (G + I)	38	65	162 (28%)	178 (31%)	161 (30%)	89 (15.4%)	155 (27%)	NR	Higher BMI and inflammatory markers were associated with AKI and RRT requirement.
Cummings [28]	257 (I)	33	62	37 (14%)	49 (19%)	NR	79 (31%)	203 (79%)	101 (39%)	CKD had a univariate HR of 1.5 for in-hospital mortality
Argenziano [29]	1000 (G + I)	40	63	137 (13.7%)	233 (23.3%)	288 (28.8%)	117 (11.7%)	233 (23.3%)	211 (21.1%)	78.0% of patients in ICU developed AKI; 35.2% of patients in intensive care units required RRT
Gupta [30]	2151 (I)	35.2	60.5	280 (12.6%)	484 (22%)	921 (43%)	432 (20%)	1494 (67.4)	784 (35%)	A score of 4 on renal component of SOFA score was associated with OR of 2.5 for 28 day mortality
Chan [7] ^a^	3235 (G + I)	42.3	66.5	323 (10%)	461 (17.4%)	1404 (46%) Stage 1: 16% Stage 2: 9% Stage 3: 21%	280 (20%)	NR	NR	Patients with AKI were older and more likely to have HTN, CHF, DM, and CKD. Independent predictors of AKI included CKD, systolic BP and potassium at baseline. Mortality of patients with AKI was 41% overall, and 52% in ICU. Adjusted OR for death was 20.9 for ICU-AKI vs no AKI.
Fisher [11] ^a^	3345 (G + I)	47	65	409 (12%)		1904 (57%) Stage 1: 50% Stage 2: 20% Stage 3:30%	164 (5%)	624 (18%)	775 (23%)	Compared with patients without COVID-19 and with historical controls, patients with COVID-19 had a significantly higher incidence of AKI and were more likely to require RRT
Hirsch [10] ^ba^	5449(G + I)	39	64	NR	949 (17.4%)	1993 (36.6%)	285 (5.2%)	1190 (21.8%)	888 (16.3%)	89.7% of patients on mechanical ventilation developed AKI compared to 21.7% of non-ventilated patients.
Richardson [31] ^ba^	5700 (G + I)	39.7	63	268 (5%)	966 (18%)	523 (22.2%)	81 (3.2%)	320 (12.2%)	553 (21%)	186 patients (3.5%) with ESKD included.

*Legend: G*- general ward, *I*- intensive care unit, *NR*- not reported, *CKD*- chronic kidney disease, *CVD*- cardiovascular and cerebrovascular disease, *HTN*- hypertension, *WBC*- white blood cell, *BUN*- blood urea nitrogen, *ESKD*- end stage kidney disease, *RRT*- renal replacement therapy, *CHF*- congestive heart failure, *DM*- diabetes mellitus, *BP*- blood pressure, *SOFA*- sequential organ failure assessment

^a^- utilized KDIGO guidelines. ^b^- Data from the same hospital system

#### United Kingdom and France

Hospital groups from the UK and France have largely focused on patients in the critical care setting. In the study from the UK (*n* = 6143), 24% required renal replacement therapy, and of these, 95.3% required advanced respiratory support (invasive ventilation, extracorporeal respiratory support) and 71% died [6]. A smaller study from Bordeaux (*n* = 71) demonstrated higher mortality in patients with AKI, and 64% of surviving patients recovered kidney function by day 21 of follow up [24]. Similar to data from China, less than 6% of patients in both studies had CKD. A larger dataset from the UK (*n* = 20,133), however, found that 16.2% of patients with COVID-19 had CKD [26]. A prospective cohort study from Spain (*n* = 1603) demonstrated that an increased serum creatinine on arrival was a risk factor for poor outcome, whether it is present acutely or as a consequence of CKD [25].

#### United States

New York City and New Orleans, Louisiana, were 2 of the more intensely affected regions of the United States. In New York, 2 large cohorts revealed AKI rates in excess of 20%, with higher proportion of CKD patients affected (10–28%) in comparison to data from China and Europe. The New Orleans (*n* = 575), Mount Sinai (*n* = 3235), Montefiore (*n* = 3345), and Northwell (*n* = 5449) hospital groups have specifically assessed AKI and associated outcomes over 5–7 weeks, with the following findings:

1. Male sex, African-American race, and age > 50 years are associated with higher risk of AKI.
2. Presence of CKD and higher potassium levels were independent predictors of stage 3 AKI (KDIGO criteria) [7].
3. Higher rates of AKI and RRT in patients with COVID-19 in comparison to historical controls (56.9% vs 25.1% for rates of AKI) [11].
4. Patients with AKI were more likely to be admitted to the ICU, undergo mechanical ventilation, and require vasopressor support [7, 10, 11].
5. AKI developed in 90% of patients on mechanical ventilation as compared to 22% of non-ventilated patients [10].
6. Ninety seven percent of patients requiring RRT were on mechanical ventilation [10].
7. Patients with AKI had higher levels of ferritin, d-dimer, C-reactive protein (CRP), procalcitonin and lactate dehydrogenase (LDH) [9, 11].
8. Mortality in patients with AKI was 45% vs 7% in those without (ICU mortality: 52% in those with AKI vs 9% in those without) [7]. AKI in hospitalized patients is associated with significant risk for in-hospital death (incidence rate of in-hospital death: 37.5/1000 patient-days in those with AKI vs 10.8/1000 patient-days in those without) [33].
9. 43% (211/486) of patients with AKI had evidence of persistent abnormal kidney function at discharge [7].

#### Summary of outcomes

There is evidence of disparate incidence and outcomes of AKI across the 3 continents, with AKI reported in 0.5–46%. Reports from Europe and the United States describe a greater burden of co-morbid disease in association with the higher rates of AKI. Additionally, the prevalence of CKD, which is a risk factor for AKI, has not been reported in most studies from China, with a low proportion of disease (~ 2%) reported in recent studies. This could be explained by absence of baseline creatinine values and/or utilization of differing criteria for diagnosis.

AKI often develops early during hospitalization, with 37–57% of patients developing AKI within 0–4 days of admission [9, 10]. Severity of pneumonia was commonly associated with lower chance of renal recovery and lower remission of proteinuria or hematuria [22]. The highest incidence of AKI (19–90%) occurs in the critical care setting, with the majority of patients requiring RRT also supported with mechanical ventilation [10].

AKI is associated with increased length of stay and mortality [7, 13, 16, 22], and this is supported by 2 meta-analyses demonstrating AKI to be an unfavorable clinical predictor associated with high mortality [34, 35]. Development of AKI appears to be a consequence of severity of illness, and its occurrence portends a worse prognosis. Current studies demonstrate up to half of patients with AKI did not recover to baseline creatinine levels and may have persistent CKD [7, 22]. Given the lack of long-term follow-up of hospitalized patients to date, the AKI recovery rate remains unknown.

#### Mechanisms and patterns of injury

AKI in patients with CoVID-19 is postulated to occur via multiple, often co-existing mechanisms (Table 2).

**Table 2:** Patterns and associated mechanisms of acute kidney injury in patients with COVID-19

Pattern	Mechanisms of Injury
**Viral cytopathic effect**	Proteins critical for mediating cellular SARS-CoV-2 infection– ACE2, TMPRSS2, and CTSL–are highly expressed in kidney [18, 36–39]. Expression is mainly localized to the apical brush border of proximal tubular cells and podocytes [36]. Viral protein and RNA have been demonstrated at the cellular level in kidney tissue, supporting a potential role for direct viral infection in the pathogenesis of AKI [18, 38–41]. Studies have demonstrated presence of mRNA in post mortem kidney tissues and its presence may correlate with clinical outcomes [42]. However, the specificity of these reports have been questioned, and the actual presence of replicating virus in renal epithelium remains controversial [43–45], as nucleic acid tests and immunohistochemistry failed to detect the virus in kidney tissues [23].
**Hemodynamic compromise**	AKI is more common in patients requiring mechanical ventilation and vasopressor support [7, 10]. Inadequate volume resuscitation and tissue hypoperfusion may lead to AKI. Hemodynamic compromise from pulmonary embolism, right ventricular dysfunction, and myocardial injury may contribute [46, 47].
**The ARDS-AKI axis**	AKI is the most common extra-pulmonary organ injury in ARDS via mechanisms including hypoxemia, reduced cardiac output, and systemic inflammation [48, 49]. Moreover, AKI-induced lung injury may further propagate severity of disease [50]. 10/20/2020 7:10:00 PM10/20/2020 7:10:00 PM
**Glomerular injury**	Possible direct viral effect and/or cytokine induced podocyte injury, along with a genetic predisposition, may result in collapsing glomerulopathy [9, 51–56].
**Rhabdomyolysis**	Rhabdomyolysis with histologic evidence of pigment deposition in renal tubules has been demonstrated [22, 40, 52, 57, 58].

Acute tubular injury is the most common etiology of AKI based on autopsy and biopsy reports [18, 22, 23, 40, 51, 52, 59], and lymphocytic infiltration is commonly present. Proximal tubular dysfunction has been demonstrated in a subset of patients with COVID-19, with resultant hypouricemia and inappropriate uricosuria correlating with disease severity and respiratory decompensation [60]. Microangiopathic injury has been infrequently observed in autopsy and biopsy series to date [52]. Hypercoagulability is a well-recognized feature of CoVID-19, although most attention has been focused on pulmonary microangiopathy, venous thromboembolism, and stroke. Terminal complement activation (C5b-9) has been demonstrated in kidney, lung, and skin, although the mechanisms underlying complement activation remain to be clarified. It has been suggested complement and neutrophil extracellular traps generate a coagulopathic milieu leading to formation of microthrombi, thereby leading to severe manifestations of COVID-19 such as lung and cardiac injury [61]. It is intriguing that despite the susceptibility of the kidney to thrombotic microangiopathy in general, most of the microangiopathic injury in CoVID-19 has been documented in extra-renal organs [18, 62, 63].

Hematuria (27–53%) and proteinuria (36–66%) are common, with higher rates reported in patients with AKI [13, 20, 22, 64]. Proteinuria is often transient, similar to Middle East Respiratory Syndrome (MERS)-CoV, although the mechanism is not known with certainty. It may be a consequence of fever, systemic inflammation, or possibly direct viral infection of renal epithelium [3, 22]. Nevertheless, the severity of proteinuria and hematuria is associated with an increased risk of mortality in patients with CoVID-19 [13]. Hematuria is often multifactorial in origin, and detailed studies on the mechanism of hematuria are lacking.

Lastly, collapsing glomerulopathy has been reported in the context of COVID-19 infection [9, 51–54]. Not surprisingly, this lesion has developed in patients homozygous for *APOL1* G1 risk alleles. This pattern of injury is most strongly associated with viral infection, particularly HIV and parvovirus. This finding suggests the presence of a high risk *APOL1* genotype and may increase the risk for interferon mediated podocyte injury due to CoVID-19. Homozygosity for high-risk *APOL1* alleles is present in 14% of African Americans, who collectively represent 12.9% of the US population but have suffered an estimated 25.1% of U.S. COVID-19 deaths [53, 54]. It remains to be determined if infection will lead to increased rates of CKD/ESKD in this population as compared to other groups.

#### Renal replacement therapy

Reported rates of RRT also vary widely, with overall rates of 2–73% in the critical care setting [6, 7, 14, 64]. The rapid surge of patients requiring RRT has led to shortages of staffing, dialysis machines, and dialysis fluid, particularly for continuous renal replacement therapy (CRRT). At the time of writing, several states in the US are expected to experience CRRT equipment shortages during the pandemic based on mathematical models [65]. This has accelerated interest in hybrid therapies such as prolonged intermittent RRT (PIRRT) with careful titration of therapy fluid rates to minimize waste [63, 66, 67]. Protocols for on-site preparation of CRRT therapy fluid have been described by multiple groups including Vanderbilt University Medical Center, Cleveland Clinic, and Johns Hopkins Hospital [68–70]. The pandemic has helped rejuvenate the utilization of acute peritoneal dialysis (PD), and data suggest the increased peritoneal pressure in setting of ARDS does not worsen hypoxemia or respiratory mechanics [71–73]. Acute PD, however, may not be suitable for prone patients. Nevertheless, even vascular access is challenging in prone patients, although skilled operators can often still insert internal jugular catheters and novel access sites such as the popliteal vein have been reported [74]. Low copies of viral RNA are present in the effluent of both PD and hemodialysis (HD) [75, 76], but there has not been isolation of infectious virus from these fluids. Current guidelines do not recommend special decontamination of effluent, and effluent should be discarded in accordance with standard practice.

### Chronic kidney disease

While there has been extensive early reporting on the impact of CoVID-19 on the kidneys in the acute setting, and the association of worse short-term outcomes including in-hospital mortality in patients with any kidney involvement, much less has been published on the impact of CoVID-19 in patients with underlying CKD. However, prior research has demonstrated that patients with CKD, and particularly those with ESKD, have been found to have immune dysregulation and increased susceptibility for infections [77]. In addition, large national organizations such as the National Kidney Foundation have published general guidelines for the care of patients with CKD in the setting of CoVID-19 [78]. Patients with any degree of CKD, for instance, have been recommended to adhere strictly to guidelines on the importance of self-isolation, and when it becomes necessary, to use face masks in public and avoid or limit exposure to large gatherings of people.

For many patients with CKD, renin-angiotensin-aldosterone system (RAAS) blockade is a mainstay of therapy. However, there has been some controversy regarding the use of RAAS blockade in the setting of COVID-19. SARS-CoV-2, similar to the SARS-CoV-1 virus that originated in 2003, uses the angiotensin-converting enzyme 2 (ACE2) receptor for viral entry [79]. ACE2 is widely expressed in a number of tissues, including the type 2 alveolar cells of lung epithelium and renal tubular epithelial cells [79]. While the more commonly recognized ACE converts angiotensin I to angiotensin II, ACE2 converts angiotensin II to angiotensin- [1–7], which acts on the Mas receptor present in many tissues throughout the body. This pathway ultimately leads to vasodilation and the systemic reduction in inflammation, as a counter-regulatory system to the vasoconstriction induced by angiotensin-II binding to angiotensin 1 receptor (AT1R).

The use of ACE inhibitors (ACEIs) and angiotensin receptor blockers (ARBs), therefore, has come under more intense scrutiny in the face of this current pandemic [80]. Controversy surrounds the potential detrimental effect of ongoing ACEIs or ARBs use, with potential up regulation of the ACE2 receptor which could then increase the ability of the virus to enter cells. It is also possible that ACEIs help by blocking the ACE2 receptor and blocking viral entry. Over the past several months, a number of large studies have explored the potential association between ACEIs or ARBs use with adverse outcomes, including COVID-19 positivity and mortality, in detail [81, 82]. Mancia and colleagues performed a population-based case-control study in Italy and demonstrated that while ACEI and ARB use was more common among patients with COVID-19, they did not find any significant association between ACEI or ARB use with risk of COVID-19 [81]. Reynolds and colleagues conducted a retrospective review of all patients admitted to the New York University Langone Health system tested for COVID-19 from March 1 to April 15, 2020, and demonstrated no substantial increase in the likelihood of COVID-19 test positivity, or severe COVID-19, based on ACEI or ARB use [82].

Various professional societies and councils have also contributed to the debate, with the American Heart Association, American College of Cardiology, and Council on Hypertension of the European Society of Cardiology, all recommending the continued use of ACEIs and ARBs. Within the field of nephrology, investigators across several institutions jointly published a perspective emphasizing the lack of clear evidence for either benefit or risk with ACEIs/ARBs use and COVID-19 [80, 83]. Without additional and high-quality clinical trial data, clinical equipoise dictates the continued use of RAAS blockade in patients who are already on these medications for other indications.

### End stage kidney disease

Among Medicare beneficiaries, patients with ESKD have the highest hospitalization rate for CoVID-19 at 1341 cases per 100,000 [84]. Patients with ESKD who are on home dialysis therapy, either PD or home HD have been able to continue dialysis while safely self-isolating. However, there are a number of unique challenges that patients undergoing in-center HD may face. While the U.S. Centers for Disease Control and Prevention (CDC) has published recommendations to reduce the spread of this highly contagious respiratory pathogen, patients undergoing in-center HD necessarily remain in dialysis units for 3 h or longer, 3 times per week, sometimes surrounded by 20 to 30 other patients, along with dialysis staff including nurses, technicians, and nephrologists. In addition to potential lack of appropriate spacing, patients with ESKD on in-center HD sometimes present to their dialysis units with symptoms of cough and shortness of breath. Such patients may then be denied entry to the HD center and require transfer to a dedicated dialysis unit and shift specific for patients under investigation (PUIs) or those with confirmed CoVID-19.

Additionally, since the start of the pandemic, the use of telemedicine has become increasingly common for the care of patients with ESKD on home therapies [85, 86]. Indeed, the International Society of Peritoneal Dialysis has provided recommendations on management of patients receiving PD in the setting of COVID-19 [87]. Overall, the use of telemedicine has allowed for continued follow-up of patients on a monthly basis with specific outlines for how to manage suspected or confirmed COVID-19, in order to minimize exposure risk.

Current recommendations for patients receiving in-center HD include screening upon entry to their dialysis unit to evaluate symptoms (e.g., shortness of breath, cough) and objective measures such as temperature checks [88]. For patients who are cleared, they should be kept at a safe distance from one another for the duration of their HD treatment. While the use of gowns, gloves, and masks have been routinely used by HD center staff at the start and end of each HD treatment, even greater emphasis should be placed on the use of personal protective equipment and hand hygiene, including masking all patients and staff during treatment. Additionally, patients with confirmed CoVID-19 or under investigation for CoVID-19 have been isolated at the level of the HD center. Among HD centers designated to take care of such patients, PUIs and patients with confirmed cases have been dialyzed on separate shifts (typically the last shift of the day), possibly on separate days depending on patient volume at the dialysis center. Another major consideration for these patients is transportation. Public transportation and dedicated mobility transportation, upon which many patients rely to and from HD centers, may not be available to those with confirmed or suspected CoVID-19 infection.

### Kidney transplantation

CoVID-19 has posed challenges to the practice of organ transplantation. Kidney transplant recipients (KTRs) are at high risk for illness, due to chronic immunosuppression and co-morbidities [89, 90]. A significant temporal association has been observed between increase in CoVID-19 infections and reduction in overall solid organ transplantation procedures [91]. This reduction in transplantation rates has been mostly seen in kidney transplantation, even in regions where CoVID-19 cases are low.

#### Current experience

At the time of writing this review, there are currently five case series along with cohort studies from US and Europe, that have reported clinical course of KTRs with COVID-19 infection (*n* = 10–1216) [85, 89, 92–98]. Fever and cough remain the most common symptoms of presentation, although atypical initial presentation with gastrointestinal symptoms has also been reported [99]. These series show a preponderance of male recipients, with median age 51–62 years (Table 3). Duration elapsed since transplantation to presentation with viral disease is highly variable with a range of 2–13 years. Of note, in two of the series, 2 patients had evidence of COVID-19 within 3 months of transplantation [92, 94]. Most of the KTRs on presentation were on maintenance immunosuppression comprising of tacrolimus (FK), mycophenolate mofetil (MMF) and prednisone. A high proportion of patients experienced AKI (30–57%) with variable rates of RRT (5–43%). Even accounting for a sizeable number of KTRs still being inpatient at the time of publication of these case series, mortality is as high as 32% [95].

**Table 3:** Characteristics of kidney transplant recipients with COVID-19

Study	N	Female, %	Median age, years	Median Transplant age, years	Maintenance IS	AKI incidence, %	RRT, %	Mechanical Ventilation, %	Mortality, %	Salient Findings
Banerjee [92]	7	42	54	2	FK + M + S - 4 FK + M - 1 AZA + S - 2	57	43	14	14	2 patients presented within 3 months of transplantation; 6 had cessation of anti-metabolites.
Nair [85]	10	40	57	7.7	FK + M + S [7] FK + M [2]	50	10	40	30	8 had cessation of MMF/MPA. All treated with HCQ and AZT.
Columbia [93]	15	30	51	4	FK - 14 M - 12 S - 10 B - 2 AZA - 1 Leflunomide - 1	40	14	27	7	10/14 had cessation of anti-metabolite; 13 received HCQ +/− AZT (6 still hospitalized at the time of publication).
Alberici [89]	20	20	59	13	FK - 19 M - 14 S - 13 mTORi - 2	30	5	0	25	All patients had their usual transplant immunosuppression withdrawn and started methylprednisolone 16 mg or equivalent dose of prednisone, and 19/20 received antiviral therapy and HCQ; 6 patients treated with tocilizumab.
Akalin [94]	36	28	60	NR	FK - 35 M - 21 S - 34	NR	21	39	28	Withdrawal of an antimetabolite in 24/28 patients (86%). FK was also withheld in 6/28 severely ill patients (21%). HCQ was administered to 24/ 28 patients (86%). Two recent KTRs who had received ATG within the previous 5 weeks died.
Lubetsky [96]	54	30	57	4.7	FK- 52 M-52 S- 20	51	10	28	13	None of the ambulatory patients had tacrolimus reduction or discontinuation of MMF
Cravedi [95]	144	35	62	5	FK - 131 M - 111 S - 125 mTORi - 11	52	NR	30	32	Non-survivors were older, had lower lymphocyte counts and eGFR, higher serum LDH, procalcitonin and IL-6 levels.
Caillard [97]	279	35	62	5	CNI- 230 M- 211 AZA- 16 S- 202 mTORi- 34 B- 16	44	11	30	23	High BMI, fever, and dyspnea were independent risk factors for severe Covid-19 in this patient group, whereas age > 60 years, cardiovascular disease, and dyspnea were independently associated with mortality.
Elias [98] *Outcomes for patients with COVID-19 reported*	1216 *(66 patients with COVID-19)*	36	54	NR	CNI- 57 M/AZA- 61 B- 6 S- 55	42	11	22	24	Factors that were independently associated with COVID-19 in- cluded non-White race and comorbidities, including obesity, di- abetes, and asthma and chronic pulmonary disease.

Legend: *IS*- immunosuppression, *AKI*- acute kidney injury, *RRT*- renal replacement therapy, *BMI*- body mass index, *FK*- Tacrolimus, *CNI*- calcineurin inhibitors, *M*- mycophenolate mofetil/mycophenolic acid, *S*- steroid, *AZA*- azathioprine, *B*- belatacept, *mTORi*- mammalian target of rapamycin inhibitor, *HCQ*- hydroxychloroquine, *AZT*- azithromycin, *KTR*- kidney transplant recipient, *ATG*- anti-thymocyte globulin, *LDH*- lactate dehydrogenase, IL-6- interleukin 6

#### Management of immunosuppression

In-vitro studies have demonstrated that calcineurin inhibitors (CNIs) such as cyclosporine and tacrolimus strongly inhibit the growth of coronavirus via inhibition of cyclophilin and immunophilin pathways [100, 101]. It is not known whether this effect of CNIs translates into clinical efficacy against the virus, but the current recommendation is to continue the CNI in KTRs since cessation may lead to increased risk of rejection and greater use of corticosteroids [102–104].

Lymphopenia is a common sign of viral infections including coronavirus, suggesting that anti-metabolites such as MMF and azathioprine be decreased or discontinued. A systematic review from 2011 showed that, in general, there is weak evidence that reduction or cessation of MMF may increase rejection rate and graft loss [103]. As such, the risk of rejection with discontinuing the anti-metabolite may be outweighed by the potential benefit in countering infection.

Based on experience from (SARS)-CoV and MERS-CoV, steroid use has been associated with delayed clearance from blood and respiratory tract [105, 106]. If steroids are a part of maintenance immunosuppression, cessation would not be recommended, although increment in dose would not be deemed beneficial unless compelling indication exists.

#### Future directions

It is difficult to ascertain the incidence and impact of COVID-19 in KTRs based on the aforementioned data. Initial reports from our center have shown an incidence of rate of 0.67% (*n* = 20), in a cohort of about 3000 patients followed with a low threshold for viral disease testing. This probably reflects that the incidence rates might be similar to normal population, but still warrants analysis of larger cohorts. It is also encouraging to note that many outpatient KTRs with known or suspected COVID-19 infection had symptomatic resolution without requiring hospitalization [107]. There is a need, however, for development of anti-viral therapy since it appears that a subset of KTRs may especially be susceptible to the disease. While hydroxychloroquine, azithromycin and tocilizumab were employed as therapy in studies discussed, it cannot be deduced that these agents are beneficial in KTRs and trials are ongoing to ascertain efficacy. Delay in transplantation is ultimately deleterious to patients in the long term, and a concerted effort on part of transplant societies and government agencies is currently paramount in overcoming this crisis.

### Socially disadvantaged populations

It is important to note that in many settings throughout the world, rates of kidney disease and the provision of its care are defined by socioeconomic, cultural, and political factors, leading to profound disparities in kidney disease burden across social strata [108]. Similarly, CoVID-19 is disproportionately impacting socially disadvantaged individuals. Such persons are often overrepresented in low-wage, public-service professions that raise risk of exposure to CoVID-19. Moreover, they may dwell in crowded, poor-quality housing that limits ability to physically distance from others to reduce infection risk [109]. In many communities, socially disadvantaged persons may also face barriers to CoVID-19 testing, fragmented access to health care, and disruptions in social services as well as greater risk of the economic consequences of CoVID-19. We will likely find that these challenges lead to delayed presentation to nephrology care (e.g. presenting with more advanced disease) and worsening of disparities in CKD and ESKD [110].

## Conclusion

CoVID-19 has been a unique challenge to the field of nephrology. Not only has it shown to be associated with high rates and vast array of presentations of AKI, it has also overwhelmed capabilities of medical systems to provide acute and chronic renal replacement therapies. Mortality in CoVID-19 patients afflicted with AKI and those with kidney transplants is high, with a high requirement for mechanical ventilation. Importantly, the disease has also further exposed glaring disparities in care of the socially disadvantaged. As our understanding of CoVID-19 will continue to evolve, there is an impetus for innovation to overcome these obstacles and develop therapeutics to support this vulnerable population.

## Data Availability

Not applicable.

## Abbreviations

CoVID-19: Coronavirus disease 2019
SARS-CoV-2: Severe acute respiratory syndrome coronavirus-2
AKI: Acute kidney injury
CKD: Chronic kidney disease
ESKD: End stage kidney disease
BMI: Body mass index
ICU: Intensive care unit
CRP: C-reactive protein
LDH: Lactate dehydrogenase
MERS-CoV: middle East Respiratory Syndrome
CRRT: Continuous renal replacement therapy
PIRRT: Prolonged intermittent RRT
PD: Peritoneal dialysis
HD: Hemodialysis
ACE2: Angiotensin-converting enzyme 2
AT1R: Angiotensin-II binding to angiotensin 1 receptor
ACEIs: Angiotensin-converting enzyme inhibitors
ARBs: Angiotensin receptor blockers
PUIs: Patients under investigation
KTRs: Kidney transplant recipients
FK: Tacrolimus
MMF: Mycophenolate mofetil
CNIs: Calcineurin inhibitors
